# Wedge-tailed Shearwater (*Ardenna pacifica*) nesting success in human-dominated coastal environments

**DOI:** 10.7717/peerj.12096

**Published:** 2021-08-30

**Authors:** Jessica L. Idle, Chad J. Wilhite, Kristen C. Harmon, Brooke Friswold, Melissa R. Price

**Affiliations:** Natural Resources and Environmental Management, University of Hawaiʻi at Mānoa, Honolulu, Hawaiʻi, United States of America

**Keywords:** Wedge-tailed Shearwaters, Nesting success, Human-wildlife interactions, Human disturbance, Symbolic fencing, Hawaiʻi, Coexistence, Seabird

## Abstract

Many seabird populations are declining globally, but successful conservation efforts have led to population expansion of some species into human-dominated landscapes. Thus, there is an increased potential for direct human and seabird interactions for certain species in human-occupied areas, with nest-site characteristics potentially affecting the susceptibility of nests to human disturbance. We assessed the effect of human activity and nest-site characteristics on Wedge-tailed Shearwater (*Ardenna pacifica,* ʻ*ua*ʻ*u kani*) nesting success at two breeding colonies, one with human exposure and one without, located in Kailua, Oʻahu, Hawaiʻi. Human activity was measured by recording the frequency of people who entered a 5 m buffer around each nest. Nests were checked every two to three days to monitor nest success. The effect of human activity and nest-site characteristics on nesting success was determined using a variety of combinations of variables within binomial logistic regression models and AICc model selection. Nest-site characteristics among nests at both sites and human activity at the human-exposed site did not show a significant effect on nesting success. Our results suggest Wedge-tailed Shearwaters may experience some tolerance of human activity immediately around their nests—as long as burrow collapse does not occur. Given the small sample sizes and a single season of data collection, additional studies are needed to better understand the effect of human disturbance on Wedge-tailed Shearwaters. Infrastructure, such as fencing and signage, may be effective at reducing human-caused nest failure and may allow humans and disturbance-tolerant seabird species to coexist in shared coastal environments.

## Introduction

Humans interact with wildlife in complex ways, leading to both beneficial and deleterious impacts at the individual- and population-level (Frank, 2016). Promoting the coexistence of wildlife and humans consists of identifying deleterious anthropogenic impacts, and then implementing mitigation strategies to address those threats. For instance, in the Pacific, seabird populations rapidly declined in the 19th century due to adult, egg, and guano harvesting (Duffy, 2010), which was later discontinued through local, federal, and international protections. Declines were also caused by the introduction of invasive mammals to islands (Blackburn et al., 2004; Blackburn et al., 2005; Duffy, 2010; Marie et al., 2014) and human land-use and development in coastal areas (Bulleri & Chapman, 2010). Coastal-dwelling seabird populations are expected to be negatively affected by ongoing sea level rise (Reynolds et al., 2015). In Hawaiʻi over 50% of beaches are at risk of coastal hardening, the construction of sea walls or other hardened structures, as a response to beach erosion concerns (Tavares, Fletcher & Anderson, 2020).

Despite these declines, recent conservation efforts are resulting in increasing seabird population growth and expansion for some species (Brooke et al., 2018), increasing the potential for human-wildlife conflict in some coastal areas. Globally, direct human interactions with nesting birds may negatively affect breeding behaviors and success by affecting chick development (Albores-Barajas, Soldatini & Furness, 2009; Cianchetti-Benedetti et al., 2018; Gyuris, 2003), nesting success (Byrd, Moriarty & Brady, 1983; Gyuris, 2003; Meixell & Flint, 2017; Rodrigues, Jesus & Campos, 2018; Watson, Bolton & Monaghan, 2014), and stress response (Ellenberg, Mattern & Seddon, 2013; Martínez-Abraín et al., 2008; Soldatini et al., 2015). Humans may directly impact reproductive success by stepping on and collapsing burrows, which can crush adult or chick inhabitants or cause suffocation if the burrow is not excavated properly (Bancroft, 2009; Byrd, Moriarty & Brady, 1983). Additionally, human activity near nesting colonies may scare away brooding adults and impede adults returning to nests to provision their young, causing concern for abandonment or reduced fitness of offspring.

Wedge-tailed Shearwaters (*Ardenna pacifica*; Hawaiian name: ʻ*ua*ʻ*u kani*; hereafter “WTSH”) are burrow-nesters that are globally-distributed in the tropics and subtropics (Shallenberger, 1973). Adults reach sexual maturity at three to five years of age and typically lay one egg per year (Byrd, Moriarty & Brady, 1983). The WTSH nesting season typically extends from March to early January, with the nest occupied during this period by an adult(s), egg, and/or chick until the fledgling emerges, typically in November or December (Byrd, Moriarty & Brady, 1983; Pyle & Pyle, 2009). Like most seabird species, a large amount of reproductive effort is required to produce a single fledgling which may eventually join the adult breeding population (Manlik, 2019); therefore, any loss or reduction in reproductive success could have significant impacts on the population as a whole. The impacts of direct human disturbance on WTSH through pedestrian traffic or scientific research varies across the global distribution (Bancroft, 2009), but at least one study suggests that nesting colonies in developed areas are particularly vulnerable (see Hill et al., 1996).

In Hawaiʻi, the eradication of the black rat (*Rattus rattus*) and the absence of other mammalian predators on many offshore islets has resulted in persisting and reproductively successful seabird colonies (Duffy, 2010; Smith, Polhemus & Vanderwerf, 2002). Wedge-tailed Shearwaters are one of the most abundant and least cryptic seabird species found in Hawaiʻi (Pyle & Pyle, 2009), and are most abundant on predator-free offshore islets. Thus, predator-free offshore islets may serve as source populations for breeding colonies on the main islands. Human access to many of the predator-free offshore islets is completely restricted or limited, but seabird colonies on the coasts of the main Hawaiian Islands are often located in human-dominated areas, or areas of high recreational or developmental value, such as beach parks. These colonies often occur in low numbers compared to offshore islet colonies, suggesting that humans may be impacting nesting success or otherwise inhibiting colony expansion.

The impacts that human disturbance may have on WTSH are not well understood, particularly for a moderate level of human disturbance, defined as the presence of humans in or near a colony, rather than a high level of disturbance through direct interactions, like handling (Carey, 2011; Saffer et al., 2000; Vertigan et al., 2012), or low level of disturbance with little to no human interactions. Moderate human disturbance to WTSH in beach parks or other human-use areas may increase the risk of human-induced burrow collapse, directly reducing nesting success (Bancroft, 2009), or increase stress to the nest occupants, indirectly reducing nesting success (Soldatini et al., 2015). The purpose of this study was to determine whether nesting success of WTSH colonies was lower at a site with a moderate level of human disturbance compared with a site with little to no human disturbance. Further, nest-site characteristics such as vegetation cover (Good, 2002), and cavity height, depth, medium and location for cavity-nesters (Mejías et al., 2017) have been documented to affect the nesting success of other seabird species. The dimensions of burrows, the medium within which the nest is made, and the vegetative cover above the nest could affect susceptibility to human disturbance, whether it be direct or indirect. Therefore, the effect of various nest-site characteristics of WTSH nests were assessed in addition to human disturbance metrics to account for potential differences between sites. Wedge-tailed Shearwaters can be used as a model species to study human and burrow-nesting seabird interactions given their global and local abundance in coastal areas.

## Materials & Methods

### Site descriptions

**Figure 1:**
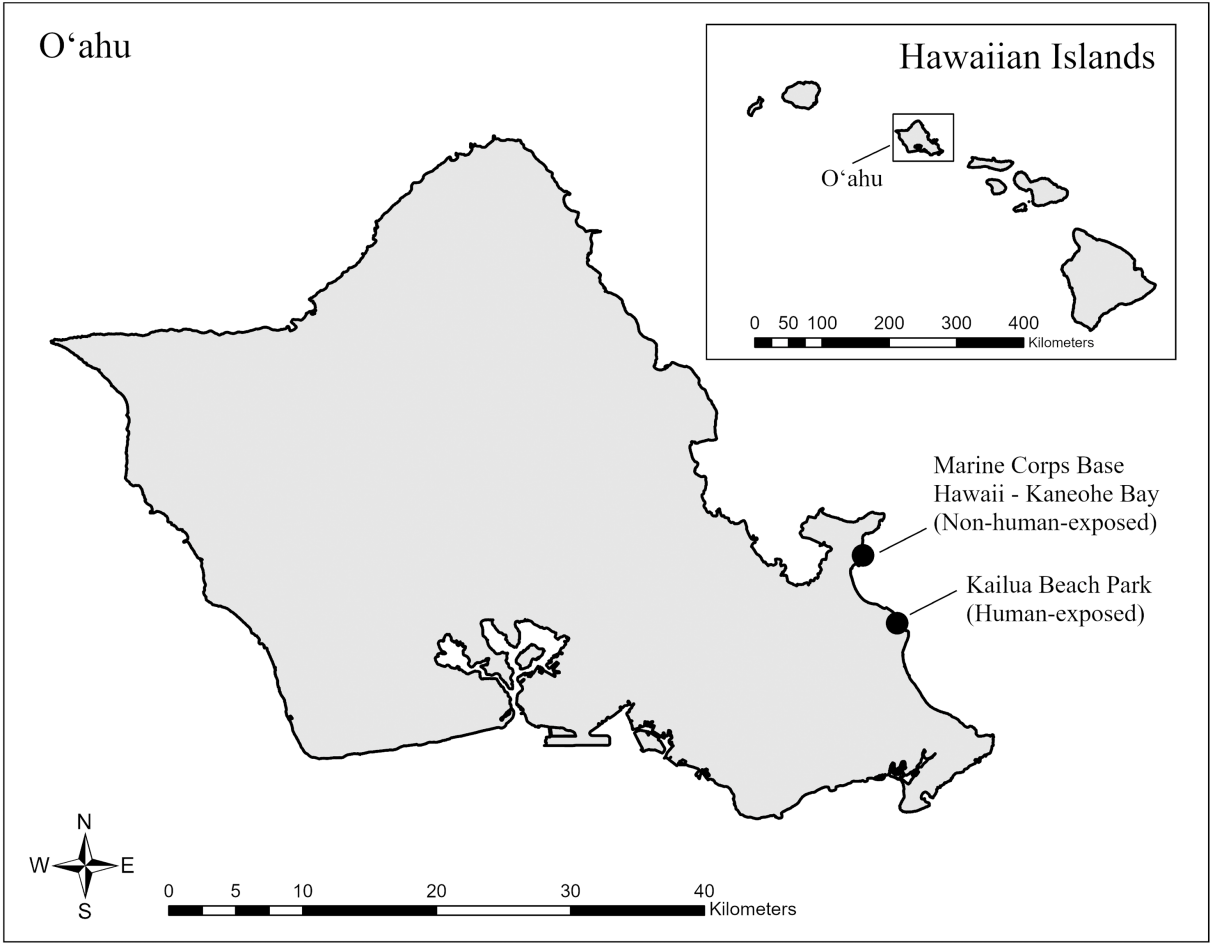
Study site locations on the island of Oʻahu, Hawaiʻi, USA. Both study sites (indicated by black circles) are located on the windward (eastern) side of Oʻahu, on opposite ends of Kailua Bay. The Marine Corps Base Hawaii–Kaneohe Bay is a federally-managed site on the north-west end of Kailua Bay that is restricted in access, while Kailua Beach Park is managed by Honolulu City and County as a popular recreational site on the south-east side of Kailua Bay.

Two nesting sites in close proximity in Kailua, Oʻahu, Hawaiʻi, USA (Fig. 1), were compared in this study, one with limited human disturbance, Marine Corps Base Hawaii - Kaneohe Bay (MCBH-KB; hereafter “non-human-exposed site”), and one with a moderate degree of human disturbance, Kailua Beach Park (KBP; hereafter “human-exposed site”). The military base is located on a peninsula on the east side of O‘ahu, positioned on the north end of Kailua Bay with nesting occurring on the eastern shoreline of the Nu‘upia Ponds Wildlife Management Area on the south side of the base. In the year of this study (2018), 912 nests were checked for occupancy during nesting surveys; 453 of which were actively occupied (L. Bookless, 2021, pers. comm.). Wedge-tailed Shearwaters nest along the coastal strand in sand with native coastal vegetation, and around the sides and rim of an oval-shaped berm that previously served as a moving target range. The area is restricted access, except for MCBH-KB Environmental Division staff, Military Police, and sponsored volunteers and researchers.

Kailua Beach Park is a Honolulu City and County recreational park on the south side of Kailua Bay that supports many forms of recreation and is a popular spot for dog walking and windsurfing. Kailua Beach Park is one of Oʻahu’s most popular beaches for residents and tourists. Between the beach and the parking lot is a strip of partially native coastal strand vegetation that serves as habitat for nesting seabirds. The beach park was suspected to host a small breeding population, perhaps colonized by individuals from nearby offshore islet source colonies, but before this study, surveys had not taken place to determine the size or state of the colony. A resident who lives near KBP installed signs on small wooden stakes connected by twine around the vegetation to warn visitors of the presence of nesting seabirds.

Most Oʻahu WTSH colonies occur on offshore islets, with few occurring on the main island of Oʻahu itself. Further, colonies on the main island are typically small, and some occur in isolated areas with limited access. Therefore, only two colonies were included in this study due to their accessibility and proximity. Some predator control methods are employed at the non-human-exposed colony, while none are known to occur at the human-exposed colony, however, it was assumed for this study that similar predator levels were present at both sites. Yet, there was the potential for additional predator threats at the human-exposed colony, such as off-leash dogs.

### Data collection

*Nest Surveys.* The study took place from August 2018, after the WTSH egg-laying period, until mid-December 2018, at the end of the typical fledging period (Byrd, Moriarty & Brady, 1983). Active burrows were identified by inserting an infrared ‘burrowscope’ (Faunatech Austbat) into each burrow to examine burrow contents (Boland & Phillips, 2005; Dyer & Hill, 1991; Dyer & Aldworth, 1998; Hamilton, 1998), and were categorized as egg, chick, adult, or empty. A complete census of all active nests was completed for the human-exposed site, resulting in a sample size of 25 active nests. As 482 active nests were identified at the non-human-exposed site, a subset of nests were randomly selected to obtain a similar sample size to the human-exposed site. Nests were chosen by surveying all of the nests within 5 m^2^ plots, which were randomly selected from a numbered grid generated using a Geographic Information System (GIS). New plots were randomly selected until at least 25 active nests were identified, resulting in a final sample size of 29 active nests that were monitored throughout the nesting season at the non-human-exposed site.

Nest occupancy was determined every two to three days. For nests classified as burrows, a burrowscope was used to determine occupancy. If the burrowscope could not be used, hand checks were conducted by inserting an arm into the burrow (Smith, Polhemus & Vanderwerf, 2002). Nests not classified as burrows could be visually checked by looking through the vegetation. Nests were classified as ‘occupied’ if an alive adult, adult and chick, or chick were found in the burrow. Nests were classified as ‘failed’ if the nest was empty for three consecutive surveys, found with an egg but no adult, or found with a carcass or other signs of predation such as feathers, blood, predator fecal matter, predator footprints, or uncharacteristic drag marks. Once a nest was unoccupied for three consecutive surveys the cause of nest failure was determined, if possible, and surveys were discontinued for that nest. Nests found unoccupied after November 15th (the beginning of the historical fledging period) were assumed to have fledged, unless observations indicated otherwise (*i.e.,* feathers, blood, or carcass indicating predation). This work was conducted under a University of Hawaiʻi at Mānoa Institutional Animal Care and Use Committee protocol (IACUC; #18-2862), State of Hawaiʻi Protected Wildlife Permit (#WL19-10), and City and County of Honolulu Department of Parks and Recreation Facility Permit (#172853).

*Human Disturbance.* Human presence near nests was recorded as a measurable proxy for direct human disturbance to nesting WTSH. These human surveys were conducted at both sites to confirm the assumption that there was no human disturbance at the non-human-exposed site, and to quantify the amount of disturbance that occurred at the human-exposed site. Prior to each nest occupancy survey at each site, the researcher positioned themselves at a spot in which all nests could be observed at the same time, but was far enough away to not disturb the colony or the people around the colony. From this position, a 5 min survey was conducted to record the number of people that passed within 5 m of each burrow. Though different activities (e.g.: walking, running, standing, shouting, walking with a dog) could cause more or less disturbance to nesting individuals, for simplicity, human activity was quantified simply as the number of people that were present near the nest within the survey period. To account for variation in human activity at different times of day, nest visits and human presence surveys were conducted within a randomly-selected 5-hour block of time between the hours of 05:00 h and 20:00 h when humans were expected to potentially be accessing the sites. Nests were surveyed for occupancy and human disturbance during the morning (5:00–10:00 h; *n* = 10), afternoon (10:00–15:00 h; *n* = 6), and night (15:00–20:00 h; *n* = 4). For analysis, the number of people that walked within 5 m of each nest was summed across all human surveys to produce a cumulative number of detections for each nest. This work was given an exemption by the University of Hawaiʻi at Mānoa Institutional Review Board (IRB) since it did not include the collection of identifiable information for individuals.

*Nest-site Characteristics.* We collected data for each nest upon discovery to account for potential differences in nest-site characteristics between the human-exposed (*n* = 25) and non-human-exposed (*n* = 29) sites and individual nests. Within a 1 m radius of each nest, using the nest as the center point, we recorded the percent cover of each plant species as <1%, 5%, 10%, 25%, 50%, 75%, or 100%. Total vegetation percent cover was then calculated by summing the percent cover of each species present within the 1 m radius plot. Plant species that occur at each site were categorized into short (<0.5 m), medium (0.5–2 m), and tall (>2 m) categories, with naupaka (*Scaevola taccada*) recorded as the only medium-height plant species. Naupaka is a dominant plant that commonly occurs in coastal areas in Hawaiʻi with dense foliar cover and intertwining branches that may serve as a natural barrier to humans or large mammals. Therefore, the percent cover of naupaka was assessed and included as a covariate explaining the probability of nest success. Each nest was also categorized by nest-type as either a burrow (*i.e.,* underground with an opening to the surface) or an indentation (*i.e.,* above ground, perhaps under taller vegetation), and the distance from the nest to the nearest pathway was recorded (m) for the human-exposed site in which paths were present.

### Data analysis

All data analyses were conducted using RStudio (R Core Development Team, 2021). To assess variation in the probability of nesting success and account for potential differences in nest-site characteristics and human disturbance metrics, several binomial generalized linear models (GLM) were built with biologically-relevant combinations of parameters including site, total percent vegetation cover, naupaka percent cover, nest-type, human detections, and distance to path. Two sets of models were built, one set for assessing the effect of the colony-wide anthropogenic disturbance level on nesting success between the human-exposed and non-human-exposed sites, and one set for assessing the effect of nest-site characteristics and variation in human disturbance metrics recorded for individual nests on nesting success within the human-exposed site only. Nests from the non-human-exposed site were excluded from the second set of models since all nests had no recorded human presence, and therefore the effect of variation in individual nest disturbance on the probability of nesting success could not be assessed for that site.

We compared each set of candidate models using sample size corrected Akaike’s Criterion (AICc) values (Akaike, Petrov & Csaki, 1973), with tables created using the ‘AICcmodavg’ package (Mazerolle, 2020). The most parsimonious model was chosen for inferences where multiple models were within two ΔAICc of the top-ranked model and fit the data better than the null model. The chosen model with the best fit was then further assessed for model fit using the Hosmer-Lemeshow goodness of fit test (Hosmer Jr & Lemeshow, 1980) with the R package ‘ResourceSelection’ (Lele, Keim & Solymos, 2019). We assessed the significance of categorical covariates in the model of best fit with a likelihood ratio test using the R package ‘lmtest’ (Zeileis & Hothorn, 2002). Lastly, we conducted a power analysis to determine the likelihood of our best fit model finding significance between sites given a sample size of 25 and using the probability estimates of nesting success at both sites.

## Results

A total of 20 human presence surveys were performed at each site over four months. The mean number of human detections at any given nest at the human-exposed site was 37 per hour (SD = 19.6, range = 0–72). At the human-exposed site, one nest failed due to burrow collapse which could have been caused by heavy rain or pressure applied on top of it, perhaps due to human traffic. The nest was less than two meters away from a path and had a moderately-high cumulative number of human detections near it (*n* = 62), compared to other nests at this site. One nest was predated, confirmed by the observation of a decomposing chick near the burrow entrance beside a path. Six nests were found empty early in the season with no signs of predation or burrow collapse. One nest was obviously stepped on, with the top of the burrow collapsed inward and a stick that had previously been resting on top of the burrow snapped in half and protruding into the collapsed burrow; however, the chick was found alive and tucked into a remaining shallow remnant of the burrow and was later assumed to have successfully fledged from the nest.

At the non-human-exposed site, two chicks were found dead outside their burrows, likely due to abandonment or predation, though neither carcass was damaged in a way that would indicate they were predated. Two nests were found empty early in the nesting season, with no indication as to occupant outcome. One chick perished most likely due to intra-specific competition-induced abandonment after it moved into another nearby nest, and one chick perished due to burrow collapse likely caused by heavy rains that occurred the day prior.

### Between-site comparison

**Figure 2:**
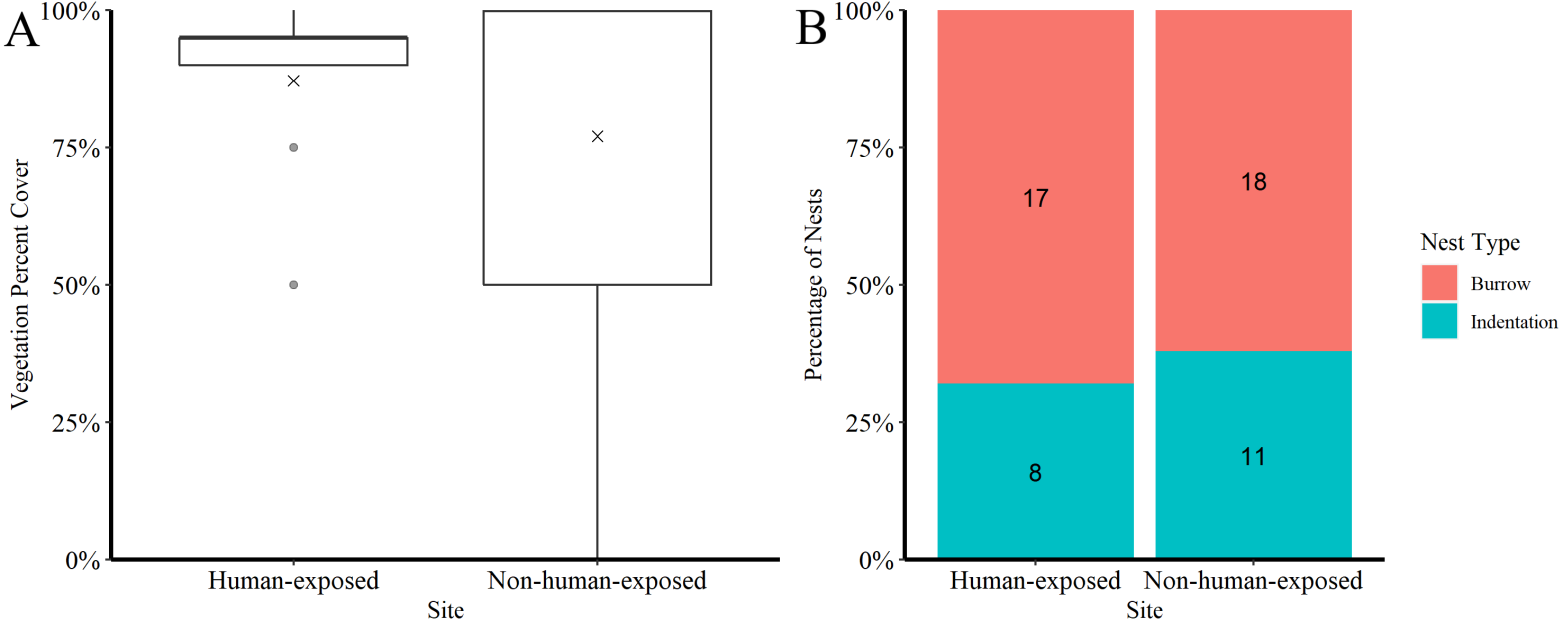
Distribution of vegetation percent cover and nest type for nests at the human-exposed and non-human-exposed sites. (A) The distribution of vegetation percent cover recorded for each nest at the human-exposed site (*n* = 25) and the non-human-exposed site (*n* = 29), with the ‘x’ symbol indicating the mean value for each site. (B) The distribution of nest types are displayed as the percentage of nests that were burrows and those that were indentations out of the total number of nests monitored at each site with the number of nests that were in each category listed in the center of each stacked color bar.

**Figure 3:**
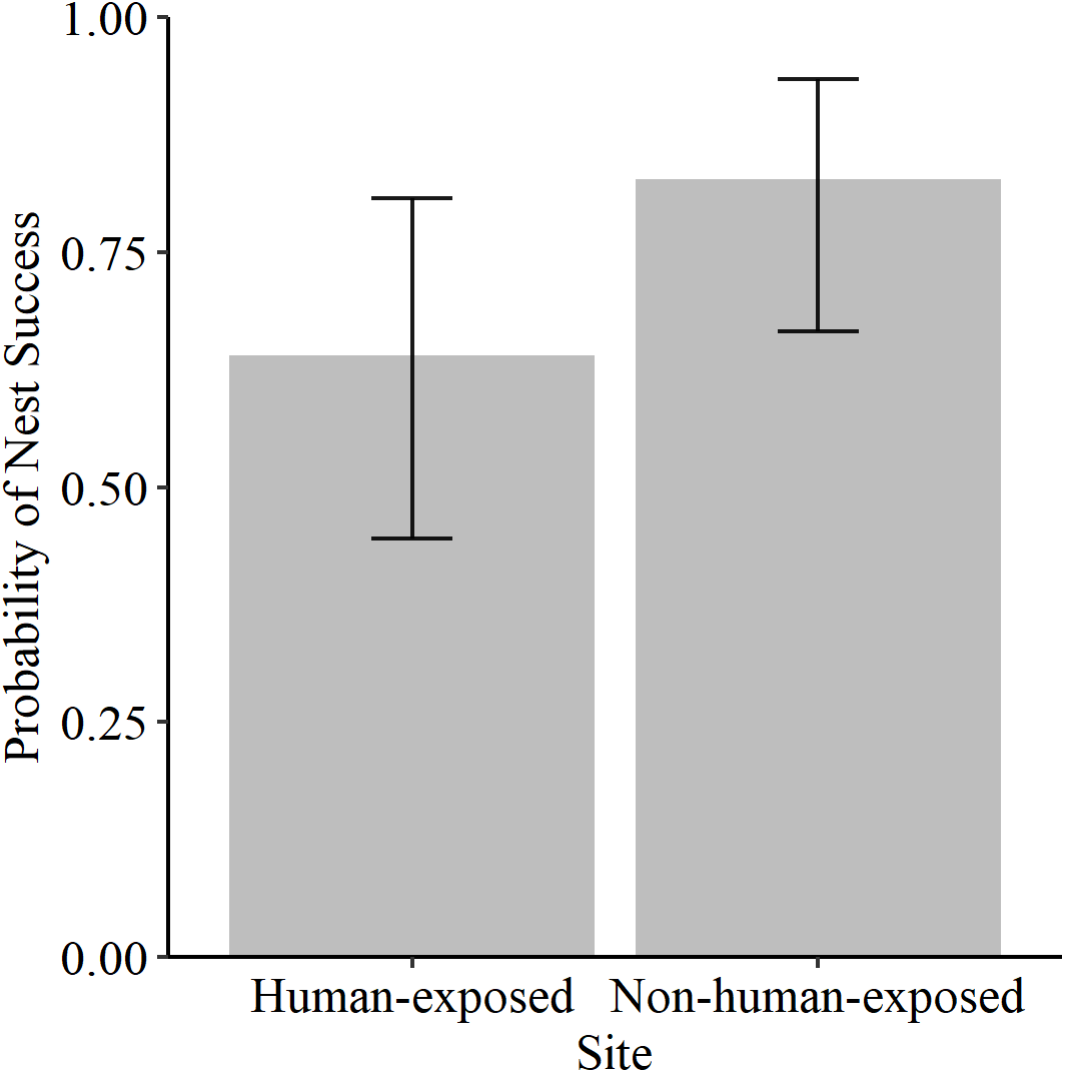
Estimated probabilities of nesting success for each site. The probability of nesting success for each site estimated from a binomial generalized linear model for the human-exposed (*n* = 25) and the non-human-exposed site (*n* = 29) with error bars representing 95% confidence intervals.

Percent cover of vegetation was similar between the two sites (Fig. 2A), as was the proportion of nests that were burrows and those that were indentations (Fig. 2B). The observed nesting success proportion was 0.67 at the human-exposed site and 0.83 for the non-human-exposed site. Comparing nesting success between sites, the best fit model (Table 1) including only ‘site’ showed no evidence for lack of fit (*p* = 1.00). Using this model, it was estimated that the nesting success probability was 0.64 (*n* = 25, 95% confidence interval = 0.45–0.81) for the human-exposed site and 0.83 (*n* = 29, 95% confidence interval = 0.67–0.94, Fig. 3) for the non-human-exposed site. However, a likelihood ratio test indicated that the ‘site’ parameter was not statistically significant (*p* = 0.12). The power of the best-fit model was estimated to be 33%.

**Table 1:** Between-site and human-exposed site model ranking. Models were built using various combinations of nest-site characteristic parameters including total vegetation percent cover, naupaka percent cover, and nest type, and human activity parameters including distance to path and human detections. (A) The five models of best fit for a between-site model comparison of nest-site characteristics and site parameters are reported (*n* = 53). (B) The five models of best fit for a model comparison of nest-site characteristic and human activity parameters for the human-exposed colony (*n* = 24) are reported. Models are ranked using Aikakes Information Criterion corrected for small sample sizes (AICc). Model comaprison metrics include the number of model parameters (K), the difference in AICc from the top model (ΔAICc), and the Aikake model weight (*w*_*i*_).

Model	df	AICc	ΔAICc	w_i_
**A–between sites**				
Site	2	63.57	0.00	0.21
Site + Nest Type	3	63.64	0.08	0.20
Null	1	63.88	0.31	0.18
Nest Type	2	64.27	0.70	0.15
Site + Total Percent Cover	3	65.77	2.20	0.07
**B–human-exposed site**				
Null	1	32.73	0.00	0.24
Human Detections + Distance to Path	3	33.42	0.70	0.17
Distance to Path	2	33.45	0.72	0.17
Nest Type	2	34.73	1.99	0.09
Human Detections	2	34.73	2.00	0.09

### Human-exposed site

At one nest at the human-exposed site, not all nest-site characteristics could be collected, though the outcome of the nest could be determined, so the sample size was reduced for nesting success analyses (*n* = 24). Within the human-exposed site, the null model was the best fit model (Table 1). The model containing all parameters (human detections, distance to path, and nest type) had the largest change in AICc from the null model; however, the next four best fit models within two AICc of the null model included each of the parameters individually.

## Discussion

### Between-site comparison

Wedge-tailed Shearwater nesting success probabilities were not significantly different between the human-exposed colony and the non-human-exposed colony, and nest-site characteristics, including naupaka cover, overall vegetation cover, distance to path, and burrow type were not predictive of nesting success. The estimated probability of nesting success was lower at the human-exposed site compared to the non-human-exposed site, but the effect was not significant. A power analysis revealed that low sample sizes reduced our statistical power, potentially reducing the ability to see the effect of human impact on nesting success. Indeed, the covariate ‘site’ appeared in the two top models, explaining more variation than the null model, indicating that there may be a difference in nesting success between sites that our analysis was not sensitive to.

### Human-exposed site

In our study, human activity and nest-site characteristics were not shown to significantly impact WTSH nesting success. Our sample size was low, however, due to a relatively low number of nesting pairs at the human-exposed study site which may have limited our ability to adequately discern factors that impacted nesting success. To address this, future studies should include more nests along a gradient of human activity. Our study adds to a growing body of contradictory literature in which some studies found significant impacts from humans on avian nesting success (Hill et al., 1996; Meixell & Flint, 2017; Rodrigues, Jesus & Campos, 2018), while others found none (Cianchetti-Benedetti et al., 2018; Gyuris, 2003). We observed only one instance of burrow collapse at the human-exposed site out of 25 nests (4%). Though there were less nests at this site compared to other studies, less nests collapsed during our whole study period compared to 15 collapsed nests and 21 damaged nests that were observed during a 6-hour walking survey in Australia (Bancroft, 2009). Investigators may have impacts on hatching success, chick growth, and survival through their activities (Bancroft, 2009; Carey, 2009; Soldatini et al., 2015), but in this study, the investigator did not cause any instances of burrow collapse, which can be common when traversing colonies. Furthermore, the use of the burrowscope likely reduced investigator-induced stress, though stress was not monitored.

The nesting success rates observed in our study were similar to those reported for the 2019 nesting season on nearby offshore islets Popoia and Mokulua Nui, where human interactions are limited by symbolic fencing and signs (Division of Forestry and Wildlife Oʻahu Branch, unpublished data). Similar to previous studies (*e.g.*, Byrd, Moriarty & Brady, 1983), our study suggests that visual barriers may be adequate to reduce nest failure caused by burrow collapse. The results of this study may have been different had a community member at the human-exposed site not placed signs and twine around the nesting areas at the beach park, potentially reducing human intrusion into the nesting area. It is likely that the tall vegetation and visual fencing put up by residents may have reduced the number of burrow collapses, indicating the potential for visual barriers as a mitigation tool. In the spring of 2021, in part as a result of this study, semi-permanent post-and-rope fencing and educational signage were installed at the human-exposed Kailua Beach Park site. We recommend similar steps at sites where managers wish to support seabird populations nesting in public coastal areas. Informative signs and public education campaigns in areas with nesting seabirds may also be effective in reducing negative human-wildlife interactions.

### Additional threats to Wedge-tailed Shearwaters

Non-human threats must also be addressed to increase the nesting success of seabirds in human-dominated areas. For example, predator control likely increases nesting success for WTSH and other native ground-nesting species (Blackburn et al., 2004; Harmon, Wehr & Price, 2021a; Jones et al., 2016; Marie et al., 2014; Vanderwerf et al., 2014). Predators were not monitored during this study but were assumed to be similar for the two study sites based on their proximity. Indeed, similar incidence of confirmed predation events occurred at each site. Some mongoose and cat traps were deployed at the large non-human-exposed site, and no predator control was conducted at the small human-exposed site. Further, we measured nesting success as the proportion of eggs that produced an individual that reached fledging, however, fledglings, particularly those of the order Procellariiformes (which includes WTSH) face many threats after they leave the burrow. Artificial lighting is known to decrease fledging success after individuals leave the burrow due to fallout, and should be minimized near seabird nesting colonies during the mating, nesting, and fledging seasons to minimize fallout in adults and fledglings (Friswold et al., 2020; Rodríguez et al., 2017; Telfer et al., 1987; Troy Jr et al., 2013). Fallout is the act of birds getting disoriented by lights, and becoming grounded, which may result in death due to dehydration, collision by vehicles, or predation. Even once individuals leave their terrestrial breeding grounds, light pollution at sea and bycatch through long-line fishing poses a significant threat to fledging and adult seabirds (Belda & Sanchez, 2001; Clay et al., 2019; Nel, Ryan & Watkins, 2002).

The time of origin of the human-exposed colony is not well known, but the colony at the military base has been documented since at least 1996, when 25-30 WTSH were identified, and surveyed regularly since 2008 (L. Bookless, 2021, pers. comm.). Vehicle access restrictions near the colony in 1998 resulted in the natural restoration of the coastal vegetation and an increase in nesting WTSH (L. Bookless, 2021, pers. comm.). The substantial increase in colony size since this time suggests large areas of habitat with limited access, such as military bases, may sometimes serve as protected areas for certain species that benefit from limited human impacts and conservation reliant species that need regular funding and management to maintain stable populations (Stein, Scott & Benton, 2008). However, disturbances at a military base may be different than those experienced at a recreational park, particularly in regards to noise, such as the use of jets, helicopters, and weapons near seabird colonies, as well as bright lights. This suggests that colonies in human-dominated areas may be limited not by direct interactions themselves, but by limited habitat availability for nesting. Thus, restoration of coastal strand vegetation, coupled with visual barriers, may be effective in increasing seabird populations in the main Hawaiian Islands for disturbance-tolerant species (akin to Lafferty, Goodman & Sandoval, 2006).

Stress indicators such as increased heart rate (Ellenberg, Mattern & Seddon, 2013) and energy expenditure (Regel & Pütz, 1997), as well as behavioral changes (Holmes, 2007), may be useful in assessing the response of seabirds to human proximity. Future studies may incorporate measurements of stress indicators to determine if there are any potential long-term effects of human disturbance on the overall health of chicks and nesting adults. In addition, follow up studies are needed that examine the impact of human activity on WTSH, specifically during times when adults are scouting and provisioning at nests. Disturbance during this time could cause adults to wait to leave the nest, or take longer to come back, and in both instances increase the amount of time between feedings for chicks. Similarly, human activities near colonies varied and were not recorded in this study, but variations in seabird responses to yelling, running, dog walking, and jumping compared to quiet walking or standing near the colony could also glean interesting results. Long-term studies of these colonies are needed to assess the potential for colony growth and recruitment outcomes for birds fledged from this site.

## Conclusions

Overall, this study provides evidence that WTSH may experience some level of tolerance to human disturbance, though additional studies with larger sample sizes are needed to better understand the effect of human disturbance on nesting success. The nesting success proportions observed between human-exposed and non-human-exposed areas suggests populations of WTSH are likely to continue to expand into human-dominated areas with available habitat in the main Hawaiian Islands. In the Anthropocene, global biodiversity is declining (Barnosky et al., 2011; Lewis & Maslin, 2015), which is increasing the need for sustainable co-existence models with native species (Price & Toonen, 2017; Winter et al., 2020) - particularly in coastal communities likely to be impacted by sea level rise (Harmon et al., 2021b). Seabird species that tolerate some level of human interaction offer the opportunity to increase or sustain native biodiversity while simultaneously allowing for human use of coastal ecosystems (Samia et al., 2015; Yorio et al., 2001).

Several management recommendations are regularly used for managing seabird species in coastal environments, including conducting regular predator control, establishing dog leash mandates, and reducing light pollution during breeding periods. In addition, community education is essential to increase awareness, transform public attitudes towards seabirds, and to garner support for promoting breeding productivity (Blanchard & Nettleship, 1992). Educational signage and visual barriers may reduce human impacts on seabirds nesting in areas with high potential for human disturbance (Duffy, 2010; Medeiros et al., 2007). Further, visual barriers and outplanting of native coastal plants are likely to facilitate the restoration of coastal habitat, increase sand retention, and reduce impacts from sea level rise (Duarte et al., 2013; Nordstrom, Lampe & Vandemark, 2000) in addition to creating more habitat for coastal ground-nesting seabirds. Seabird species that express some tolerance to human activity may thus be a core component of co-existence models in which we shift human-dominated landscapes into spaces where both native species and humans can thrive into the next century.

##  Supplemental Information

10.7717/peerj.12096/supp-1 Supplemental Information 1 Dataset of nest-site characteristics and nesting success of each nest at both sites Each nest has a unique alpha-numeric code in the “Nest ID” column. Each nest is then categorized by “site” (KBP = human-exposed, MCB = non-human-exposed), “success” (0 = failure, 1 = success), and “nest.type” (B = burrow, I = indentation). The total vegetation cover (“Total.Cover”) and naupaka percent cover (Naupaka.Cover) are represented as proportions and are recorded for each nest. Lastly, the total number of human detections recorded for each nest during the entire study period are recorded under “Human.Detections”, and the distance from the nest to the nearest path (m) is recorded for each nest (“Dist.Path”).

10.7717/peerj.12096/supp-2 Supplemental Information 2 R code for data analyses Compare the two sites and the effect of nest-site characteristics on nesting success.

## Additional Information and Declarations

### Field Study Permissions

The following information was supplied relating to field study approvals (i.e., approving body and any reference numbers): Field experiments were approved by the State of Hawaiʻi (State of Hawaiʻi Protected Wildlife Permit no. WL19-10) and the City and County of Honolulu Department of Parks and Recreation (City and County Facility Permit #172853).

### Data Availability

The following information was supplied regarding data availability: The raw measurements of human detections and nest-site characteristics and the code in R for analyses that compare the two sites and the effect of nest-site characteristics on nesting success are available in the Supplementary Files.
